# Polymeric Nanoparticles Potentiate the Anticancer Activity of Novel PI3Kα Inhibitors Against Triple-Negative Breast Cancer Cells

**DOI:** 10.3390/biomedicines12122676

**Published:** 2024-11-24

**Authors:** Suhair Sunoqrot, Samah Abusulieh, Dima Sabbah

**Affiliations:** Department of Pharmacy, Faculty of Pharmacy, Al-Zaytoonah University of Jordan, Amman 11733, Jordan

**Keywords:** PI3Kα, polymeric nanoparticles, protein kinases, TPGS, triple-negative breast cancer

## Abstract

**Background**: Dysregulation in phosphoinositide-3-kinase alpha (PI3Kα) signaling is implicated in the development of various cancers, including triple-negative breast cancer (TNBC). We have previously synthesized a series of N-phenyl-6-chloro-4-hydroxy-2-quinolone-3-carboxamides as targeted inhibitors against PI3Kα. Herein, two drug candidates, R7 and R11, were selected to be further investigated as a nanoparticle (NP) formulation against TNBC. **Methods**: R7 and R11 were entrapped in D-α-tocopheryl poly(ethylene glycol) 1000 succinate (TPGS) polymeric NPs by nanoprecipitation. Following their physicochemical characterization, the anticancer activity of the compounds and their NP formulations was evaluated in the TNBC cell line MDA-MB-231 by conducting viability, uptake, and apoptosis assays, as well as penetration assays in a multicellular tumor spheroid model. **Results**: The NPs exhibited a particle size of 100–200 nm, excellent drug loading efficiencies, and sustained release under physiologic conditions. Viability assays revealed superior potency for the NP formulations, with IC_50_ values of 20 µM and 30 µM for R7- and R11-loaded NPs, respectively, compared to the free compounds, which exhibited IC_50_ values of 280 µM and 290 µM for R7 and R11, respectively. These results were attributed to the inherent antiproliferative activity of TPGS, as evidenced by the cytotoxicity of the drug-free NPs, as well as the enhanced cellular uptake enabled by the NP vehicle, as demonstrated by fluorescence microscopy imaging and flow cytometry measurements. Further investigations showed that the NPs promoted apoptosis via a mitochondrial-dependent pathway that involved the activation of proapoptotic caspases. Moreover, the NP formulations enhanced the penetration ability of the free compounds in multicellular tumor spheroids, causing a time- and concentration-dependent disruption of the spheroids. **Conclusions:** Our findings highlight the important role nanotechnology can play in improving the biopharmaceutical properties of new drug candidates and facilitating their in vivo translation.

## 1. Introduction

Cancer is one of the most common fatal disorders, responsible for almost 10 million deaths in 2020 [1]. Despite extensive laboratory, epidemiological, and clinical studies conducted over the last decades, breast cancer incidence continues to rise [2], accounting for the highest number of diagnosed cases in 2020 [1], with more than 2 million women diagnosed in 2022 [3]. The molecular subtypes of breast cancer are classified by the expression of hormone receptors and growth factors (estrogen receptor (ER), progesterone receptor (PR), and human epidermal growth factor receptor 2 (HER2). Triple-negative breast cancer (TNBC) is characterized by the low expression of ER, PR, and HER2; therefore, it does not respond to treatments that target these receptors [4]. TNBC is more aggressive and differentiated than other invasive breast cancer subtypes, with a higher growth rate and molecular heterogeneity of tumor cells [5]. TNBC is associated with a high mortality rate, increased risk of early recurrence, and the possibility of distant metastasis [6]. The treatment of TNBC has been a major challenge due to its poor prognosis, heterogeneity, aggressiveness, and metastatic spread of the disease [7]. Available treatment options for TNBC include conventional chemotherapy, radiotherapy, surgery, immunotherapy, and novel targeted and nanoparticle-based therapies [5,6,7,8]. Out of these, chemotherapy is the most common, but it has limitations because of its high toxicity and side effects, lack of specificity, development of chemotherapeutic resistance, and poor therapeutic outcomes [8]. These restrictions can be improved by identifying potential molecular targets and developing novel targeted therapies, including kinase inhibitors.

Protein kinases (PKs) have an important role in a wide range of cellular functions, including metabolism, differentiation, survival, and cell cycle regulation. Dysregulation in the activity of PKs has been implicated in the development of autoimmune, inflammatory, and cardiovascular disorders, in addition to several types of cancers. As a result, PKs have emerged as an important target for drug development [9]. Among the different types of PKs, the phosphoinositide-3-kinase (PI3K) signaling pathway is involved in crucial cellular processes, and its activation is connected to cancer development [10]. PI3K stimulates the phosphorylation of protein kinase B (AKT), resulting in the activation of several downstream kinases, such as the mammalian target of rapamycin (mTOR), eventually promoting cell growth and proliferation [11]. The PI3K/AKT/mTOR signaling cascade is a key regulator in tumor progression, allowing the development of targeted therapies [12,13,14]. PI3Ks are classified into three classes: I, II, and III. Class I PI3Ks are heterodimers that comprise a regulatory (p85) and a catalytic (p110) subunit [15]. This class is further divided into four isoforms: α, β, γ, and δ [16]. PI3Kα is the most frequently mutated isoform in breast cancer, making it a promising therapeutic target [17]. Alterations in the PI3Kα signaling pathway have been connected to carcinogenesis, angiogenesis, and metastasis [13,18].

**Figure 1:**
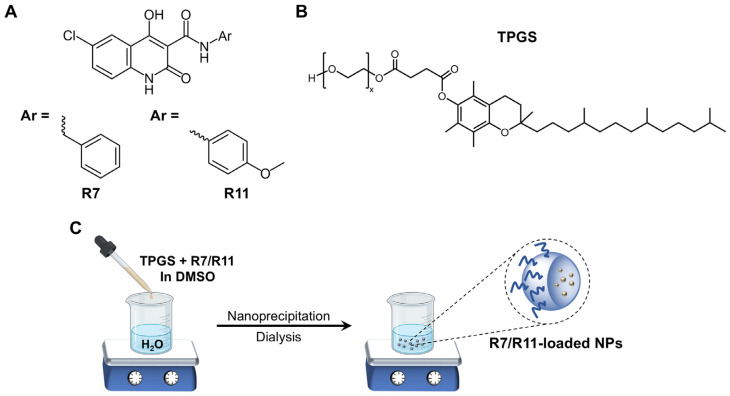
(**A**) Structure of R7 and R11 compounds; (**B**) Structure of TPGS; (**C**) Preparation of R7- and R11-loaded TPGS NPs by nanoprecipitation.

We have recently reported on the design and synthesis of new PI3Kα inhibitors derived from an N-phenyl-6-chloro-4-hydroxy-2-quinolone-3-carboxamide nucleus (Figure 1A). The series included 18 compounds that were investigated as antiproliferative agents against human colon cancer cell lines HCT-116 and Caco-2 [19]. Of note were two compounds that showed micromolar potencies against both cell lines: the N-benzyl- (R7; 15 and 14 µM, respectively) and the N-(4-methoxyphenyl)- derivative (R11; 9 and 14 µM, respectively).

Despite their promising anticancer activity, these newly developed anticancer agents suffer from poor aqueous solubility, which could limit their in vivo performance and clinical translation. Nanotechnology-based platforms offer promising strategies to enhance the delivery efficacy of anticancer agents. Nanocarriers have distinct characteristics such as nanometric size, long circulation half-lives, high drug loading capacity, active and passive targeting, controlled release, reduced toxicity, and enhanced efficacy over conventional chemotherapy [20,21]. The controlled release to precise sites and tumor-targeted delivery of these anticancer drugs by nanoparticles (NPs) improve therapeutic efficiency, limit toxicity to normal cells, and reduce side effects of chemotherapies [22]. This is mainly attributed to the passive targeting ability of nanocarriers based on the enhanced permeation and retention (EPR) effect [23]. Polymeric NPs, a group of colloidal systems with particle size less than 200 nm, have been widely explored as carriers for anticancer drugs as well as other therapeutics due to their biodegradability and/or biocompatibility [22,24]. Drug molecules can be conjugated, entrapped, or encapsulated into the polymer matrix by a variety of techniques, including emulsion-solvent evaporation, high-pressure homogenization, and nanoprecipitation [25]. Consequently, they provide a protective covering for the drugs to limit their toxicity and improve their intracellular distribution. Moreover, their minuscule size allows them to cross biological membranes and penetrate narrow capillary vessels, promoting increased uptake by tumor cells [24].

We have recently developed polymeric NP formulations for one of the lead compounds of the N-phenyl-6-chloro-4-hydroxy-2-quinolone-3-carboxamide series (R19) using D-α-tocopheryl polyethylene glycol 1000 succinate (TPGS) and Pluronic P123 polymers [26]. TPGS, an amphiphilic vitamin E derivative, has been employed as a nanocarrier for hydrophobic drugs to improve bioavailability, prolong circulation half-life, enhance cytotoxicity, and synergize with other chemotherapeutic agents. The incorporation of TPGS in R19-loaded NPs enhanced its antiproliferative and proapoptotic activity in both hormone receptor-positive (MCF-7) and triple-negative (MDA-MB-231) breast cancer cell lines. Our results supported the application of the designed TPGS NPs as a delivery system against breast cancer [26]. The purpose of the current study was to develop a polymeric nanoformulation based on TPGS for two promising PI3Kα inhibitors of the same N-phenyl-6-chloro-4-hydroxy-2-quinolone-3-carboxamide series: R7 and R11 (Figure 1). The drug molecules were entrapped in TPGS NPs, followed by physicochemical characterization. Given the aforementioned challenges of treating TNBC, the biological assays were focused on MDA-MB-231 cells as an in vitro model, providing insights into the ability of NP delivery systems to enhance the bioactivity of newly developed medicinal agents.

## 2. Materials and Methods

### 2.1. Materials

The compounds R7 and R11 were synthesized and characterized in our previous publication [19]. D-α-tocopheryl poly(ethylene glycol) 1000 succinate (TPGS) and trypan blue solution were acquired from Sigma-Aldrich (St Louis, MO, USA). Potassium bromide (KBr) and dimethyl sulfoxide (DMSO) were obtained from Fisher Chemicals (Loughborough, UK). Spectra/Por dialysis membranes with 3.5 and 12–14 kDa molecular weight cut-offs (MWCO) were procured from Repligen (Waltham, MA, USA). Ultrapure water was obtained via an EMD Millipore Direct-Q 5UV purification system (Burlington, MA, USA). Phosphate-buffered saline solution (PBS, 10×, pH 7.4) was purchased from EuroClone (Milan, Italy). Tween 80 was obtained from RCF Limited (Mumbai, India). Resazurin sodium salt (RZ) was purchased from TCI (Tokyo, Japan).

### 2.2. Preparation of R7- and R11-Loaded NPs

In this study, R7- and R11-loaded NPs were formulated using the nanoprecipitation method (Figure 1C), following our previous study [26]. The composition of the different NPs is detailed in Table 1. Briefly, the organic phase was prepared by mixing 1 or 2 mg of each R7 or R11 powder with 10 mg of TPGS in 0.5 mL of DMSO, and the mixtures were completely dissolved under sonication (Elmasonic S 40-H, Singen, Germany) for 30 min with heat to obtain clear solutions. The samples were then dropped into 5 mL of ultrapure water in a glass vial under stirring for 30 min at 400 rpm. The obtained NP dispersions were purified by dialysis (3.5 kDa MWCO dialysis membrane) against deionized water overnight, changing the water regularly, and then stored at 4 °C. Blank (drug-free) NPs were obtained following the same procedure without the addition of the drugs.

### 2.3. Characterization of R7- and R11-Loaded NPs

#### 2.3.1. Measurement of Particle Size and Zeta Potential

Freshly prepared R7- and R11-loaded NPs were characterized in terms of particle size, polydispersity index (PDI), and zeta potential by dynamic light scattering (DLS) and electrophoretic light scattering (ELS), respectively. Measurements were acquired using a Nicomp Nano Z3000 particle sizing system (Entegris, Billerica, MA, USA). Prior to each measurement, samples were diluted 10× with ultrapure water. Mean values ± standard deviation (SD) were reported from at least three different batches of each formulation. To evaluate the stability of the NPs, fresh batches were incubated in RPMI 1640 cell culture medium supplemented with 10% fetal bovine serum (FBS) for 1 h at 37 °C, followed by remeasuring the particle size by DLS.

#### 2.3.2. Determination of Drug Loading Efficiency (DL%)

The loading efficiency of R7 and R11 was measured by UV–Vis spectroscopy (UV-1800, Shimadzu, Kyoto, Japan). Aliquots from freshly prepared NPs were diluted 20× in DMSO to dissolve the NPs, and the UV absorbance of R7 and R11 was recorded at λ_max_ = 305 and 337 nm, respectively. A calibration curve was constructed for each molecule within the concentration range of 20–1.25 µg/mL in DMSO, which was employed to calculate the concentration and the total amount of each drug loaded in the NPs. Each measurement was performed in triplicate. Drug loading efficiency (DL%) was calculated based on Equation (1):

(1) $$DL\%=\frac{Actual\;amount\;of\;loaded\;drug\;(mg)}{Theoretical\;amount\;of\;drug\;(mg)}\times 100\%$$

#### 2.3.3. Fourier Transform-Infrared (FT-IR) Spectroscopy

An IR Affinity-1 spectrometer (Shimadzu, Japan) was used to scan the FT-IR spectra over the wavenumber range of 4600–650 cm^−1^. For analysis, drug-loaded NPs were first lyophilized using a FreeZone freeze dryer system (4.5 L Benchtop, Labconco, Kansas City, MO, USA) at −50 °C and 0.05 mbar. Lyophilized NPs, R7, R11, and TPGS were pressed with KBr into discs and prepared for measurement.

#### 2.3.4. In Vitro Release Studies

The dialysis method was used to evaluate the in vitro release of R7 and R11 from loaded NPs at pH 7.4 and 37 °C [26]. One-milliliter aliquots of freshly prepared NP dispersions were placed in a dialysis membrane (12–14 kDa MWCO) in triplicate. Each bag was tightly closed and individually submerged in a glass vial containing 30 mL of PBS (pH 7.4) containing 0.5% *w*/*v* Tween 80 to ensure sink condition. The vials were incubated in an orbital shaker-incubator (Biosan ES-20, Riga, Lativa) set to 100 rpm and 37 °C. Ten milliliter samples were withdrawn from the release medium at 1, 2, 4, 6, and 8 h and exchanged with an equal volume of fresh release medium. At the 24, 48, 72, and 96 h time points, the entire volume was collected and replaced with 30 mL of the release medium. The withdrawn samples were freeze-dried and then redissolved in 2 mL of DMSO to concentrate the samples. Undissolved buffer salts were precipitated by centrifugation (4000× *g* for 5 min). The amounts of R7 and R11 released were measured by UV–Vis spectroscopy based on a calibration curve of each drug absorbance versus concentration in DMSO. Cumulative release was determined as the percentage of the cumulative amount of drug released at each time point relative to the total amount of drug in the NPs. The release profiles were then constructed by plotting the average cumulative release (%) of each drug against time (h).

### 2.4. Evaluation of the Antiproliferative Activity of R7- and R11-Loaded NPs

#### 2.4.1. Cell Culture

The antiproliferative activity of R7 and R11 NPs was tested on the MDA-MB-231 human breast cancer cell line purchased from the American Type Culture Collection (ATCC, Manassas, VA, USA) and cultured in T-75 cm^2^ cell culture flasks. Cells were grown in a complete growth medium consisting of Roswell Park Memorial Institute (RPMI 1640) medium (EuroClone, Italy) supplemented with 10% (*v*/*v*) heat-inactivated fetal bovine serum (FBS; EuroClone) and 1% (*v*/*v*) penicillin/streptomycin antibiotics (100 U/mL-100 µg/mL; EuroClone). Cultures were maintained at 37 °C in a humidified 5% CO_2_ incubator. Cells were routinely sub-cultured using trypsin-EDTA solution, and the culture medium was changed every two days. To perform each experiment, cell culture flasks were used at around 80–90% confluence and 4–10 passage numbers.

#### 2.4.2. Cell Viability Assay

The effects of R7, R11, and their loaded NPs on the viability of MDA-MB-231 cells were evaluated in 2D cultures of adherent cell monolayers by means of a resazurin assay as previously described [27]. When the cells reached 80% confluence, they were trypsinized from the culture flask, centrifuged, and resuspended in a fresh culture medium. Subsequently, cells were counted using trypan blue solution and seeded in 96-well plates at a density of 10,000 cells/well overnight. After 24 h, cells (*n* = 5 per treatment group) were treated with R7 and R11 (from 20 mg/mL stock solutions in DMSO) and their corresponding NPs at concentrations equivalent to 300, 100, 10, 1, and 0.1 μM of R7 and R11 diluted in complete culture medium. Blank (drug-free) NPs were also applied at concentrations equal to the drug-loaded NPs. Doxorubicin HCl (TCI, Japan) was used as a positive control within the concentration range of 100, 10, 1, 0.1, and 0.01 μM. After treating the cells for 72 h, the media in each well was replaced with 100 μL of fresh media containing 0.015 mg/mL resazurin sodium salt (0.15 mg/mL stock in PBS diluted 10× in the culture media). Cells were incubated for another 3 h (37 °C, 5% CO_2_), followed by reading the fluorescence intensity of the plates at 540 nm/620 nm excitation/emission wavelengths (Synergy HTX Multi-Mode microplate reader; BioTek, Winooski, VT, USA). Cell viability was calculated as the ratio of the fluorescence of each sample relative to the untreated cells and expressed as a percentage. Non-linear regression analysis of cell viability versus concentration curves was then employed to determine the half maximal inhibitory concentrations (IC_50_) values of each treatment using GraphPad Prism software version 9.

### 2.5. Cellular Uptake of Labeled NPs by Fluorescence Microscopy and Flow Cytometry

To visualize and quantify the cellular uptake of R7 and R11 NPs, coumarin 6 (C6) was loaded in TPGS NPs by replacing R7/R11 with 0.1 mg C6 during the nanoprecipitation process. For fluorescence microscopy imaging, MDA-MB-231 cells were seeded in 24-well plates at 50,000 cells/well (*n* = 3). After 24 h, free C6 or C6-labeled NPs were applied to the cells at a concentration equivalent to 1 µg/mL C6 in serum-free media for 2 h. After washing gently with PBS, 4% paraformaldehyde was added for 10 min. After, cells were washed again with PBS and imaged using the green channel of a ZOE Fluorescent Cell Imager (Bio-Rad Laboratories, Hercules, CA, USA) [27]. For quantitative measurement of cell-associated fluorescence by flow cytometry, cells were seeded as described above, and then 1 µg/mL free C6 or C6-labeled NPs were applied in serum-free media for 2 h in triplicate. After washing with PBS, trypsin-EDTA (0.1 mL/well) was added, and the cells were incubated at 37 °C for 10 min, centrifuged at 150× *g* for 5 min, and resuspended in 0.5 mL PBS. Cell-associated fluorescence was measured immediately at 488 nm excitation and 533/30 nm emission wavelengths using a BD Accuri C6 Plus Flow Cytometer (BD Biosciences, Franklin Lakes, NJ, USA) [28], with the threshold set to 10,000 events. Analysis was performed using BD Accuri C6 Plus software. Results were expressed as the fold increase in fluorescence count relative to untreated cells.

### 2.6. Evaluation of the Proapoptotic Activity of R7- and R11-Loaded NPs

#### 2.6.1. Mitochondrial Membrane Depolarization Assay

The JC-1 dye (Abcam, Cambridge, UK) was used as a probe to measure changes in mitochondrial membrane potential in MDA-MB-231 cells treated with R7 and R11, as well as their NP formulations. The assay was performed as previously described [29]. Briefly, cells were seeded in a 96-well plate at 10,000 cells/well (*n* = 3) overnight and then treated with the free drugs, drug-loaded NPs, and blank NPs at concentrations equivalent to 20 and 30 μM of R7 and R11, respectively, diluted in complete culture medium. After 24 h of incubation, 100 μL of JC-1 working solution (20 µM) was added directly to each well, and the cells were stained for 10 min at 37 °C. JC-1 forms red fluorescent aggregates inside intact mitochondria that are converted to green fluorescent monomers upon depolarization of the mitochondrial membrane during early apoptosis [30,31]. JC-1 monomers and aggregates were detected at 485 nm/528 nm and 540 nm/620 nm excitation/emission wavelengths, respectively (Synergy HTX Multi-Mode microplate reader). The results were expressed as the normalized JC-1 monomer/aggregate ratio relative to the untreated cells. For JC-1 detection by fluorescence imaging, cells were treated as described above. Following JC-1 staining, cells were imaged using the green and red channels of a ZOE Fluorescent Cell Imager (Bio-Rad Laboratories, USA).

#### 2.6.2. Caspase Activity Assay

Activation of key caspases (caspase-3, -8, and -9) in MDA-MB-231 cells undergoing apoptosis after treatment was monitored using a caspase multiplex activity assay kit (Abcam, UK) as previously described [29]. For the assay, cells were seeded in 96-well plates (10,000 cells/well) overnight. The following day, cells (*n* = 3) were treated with the free drugs, drug-loaded NPs, and blank NPs at concentrations equivalent to 20 and 30 μM of R7 and R11, respectively, diluted in a complete culture medium for 24 h. At the end of the 24 h treatment, 11 μL of each caspase (caspase-3, caspase-8, and caspase-9) were added to 2.2 mL of the assay buffer, and 100 μL of the caspase mix solution was added to each well. After incubation for 1 h, the fluorescence intensities of the fluorogenic indicators for caspase-3, caspase-8, and caspase-9 were detected at 540 nm/620 nm, 485 nm/528 nm, and 360 nm/460 nm excitation/emission wavelengths, respectively. The results were expressed as the fold increase in fluorescence intensity of treated cells relative to the untreated controls (mean ± SD).

### 2.7. Spheroid Formation Assay

The antiproliferative activity of R7- and R11-loaded NPs was further investigated by evaluating their ability to inhibit the growth of MDA-MB-231 multicellular tumor spheroids. The technique employed for spheroid formation was based on the use of ultra-low attachment plates, as previously reported [32]. For the assay, cells were seeded in round bottom ultra-low attachment 96-well plates (SPL, Gyeonggi-do, South Korea) at 5000 cells/well (200 µL/well) and incubated for 4 days, changing the medium every two days by removing 100 µL and replacing with 100 µL of fresh medium. Spheroid formation was monitored daily by viewing under a Primovert inverted microscope (Zeiss, Oberkochen, Germany) equipped with an Axiocam ERc 5s digital camera. After allowing the spheroids to grow for 4 days, the spheroids were imaged at 4× magnification and then treated with R7 and R11, their corresponding NPs, and blank NPs at 10 and 100 µM in complete culture medium. Stock solutions of the samples were prepared at double the intended concentrations, and 100 μL of the medium was replaced with 100 μL of each sample. After 72 h of treatment, the wells were imaged again to visualize the effect of the treatments on spheroid formation.

### 2.8. Statistical Analysis

All results were reported as the mean ± SD of at least three independent experiments. Statistical analysis was performed in GraphPad Prism 9 using a one-way analysis of variance (ANOVA) followed by Tukey’s post-hoc analysis, where *p* < 0.05 was considered statistically significant.

## 3. Results

### 3.1. Preparation and Characterization of R7- and R11-Loaded NPs

N-phenyl-6-chloro-4-hydroxy-2-quinolone-3-carboxamide derivatives are a newly developed class of PI3Kα inhibitors which demonstrated promising activity against human cancers [19]. However, as with many new drug candidates, these compounds suffer from poor aqueous solubility, which can limit their in vivo efficacy. We have recently shown that polymeric NPs composed of TPGS and Pluronic P123 could effectively entrap one of the newly synthesized N-phenyl-6-chloro-4-hydroxy-2-quinolone-3-carboxamide derivatives (R19) [26]. The NPs successfully delivered the drug to MCF-7 and MDA-MB-231 breast cancer cells and enhanced its anticancer activity. In this work, two additional derivatives, R7 and R11 (Figure 1A), were selected for their notable anticancer potencies and formulated in the form of NPs using TPGS as the polymeric carrier (Figure 1B). TPGS, an amphiphilic biocompatible excipient, also possesses intrinsic anticancer activity [33], which could prove beneficial in combatting aggressive forms of cancer such as TNBC.

**Figure 2:**
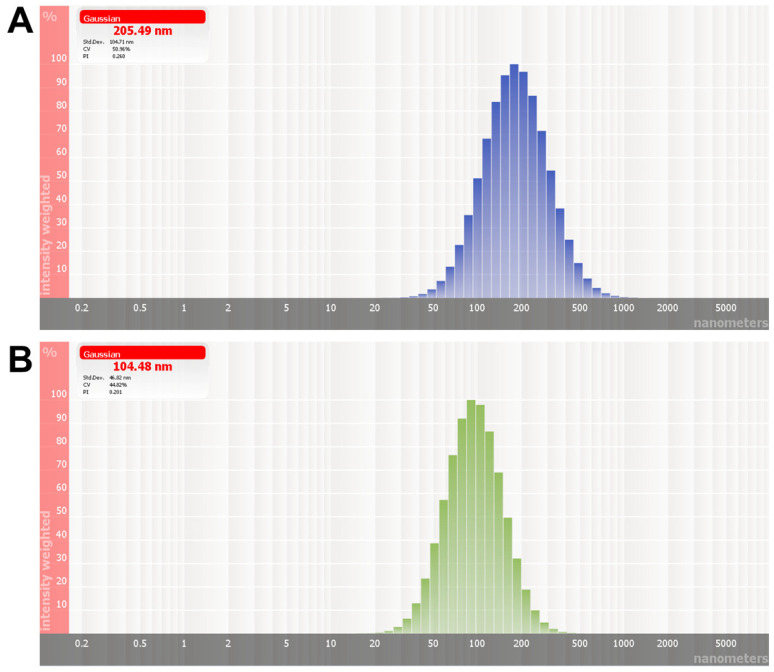
Representative intensity-weighted particle size distributions of (**A**) R7 NP2 and (**B**) R11 NP2.

R7 and R11 were entrapped in TPGS NPs by the nanoprecipitation technique (Figure 1C). The procedure involves preparing solvent and nonsolvent phases separately, followed by adding one phase to the other under moderate stirring. The solvent phase is typically composed of an organic water-miscible solvent in which the polymer and hydrophobic drug are dissolved. The nonsolvent phase is composed of water with or without surfactants or stabilizers [34]. Mixing the solvent with the nonsolvent results in the precipitation of the polymer in the form of nanospheres, entrapping the drug in the NP matrix [35]. In this study, the polymeric carrier TPGS was dissolved alongside the drug molecules in DMSO, followed by dropwise addition to the aqueous phase under stirring. After mixing to allow NP formation, the unentrapped molecules and DMSO were removed from the NP dispersion by membrane dialysis. As summarized in Table 2, both R7 and R11 were successfully loaded in TPGS NPs, with loading efficiencies between 70 and 91% depending on the initial amount of drug loaded and the type of drug. For example, R7 added at 1 mg (R7 NP1) resulted in NPs with a hydrodynamic diameter of 175 nm and a loading efficiency of 70%. Both the particle size and loading efficiency were increased when the amount of R7 was increased to 2 mg (while fixing the amount of TPGS), reaching 203 nm and 76%, respectively. The particle size of R7 NP2 was significantly greater than R7 NP1 (*p* < 0.05). However, the loading efficiencies between the two formulations were comparable. On the other hand, the two formulations of R11-loaded NPs exhibited comparable hydrodynamic diameters between 100 and 109 nm, which were significantly smaller than R7 NPs (*p* < 0.0001). Moreover, R11-loaded NPs achieved loading efficiencies of 84 and 91% for R11 NP1 (1 mg R11) and R11 NP2 (2 mg R11), respectively. The smaller diameter and higher loading capacity observed in R11-loaded NPs compared to their R7 counterparts suggest a greater affinity between R11 and TPGS. As shown in Figure 1A, the presence of an extra methoxy group in R11 could impart additional points of interaction with TPGS via hydrogen bonding, resulting in tighter packing and a greater loading capacity. All formulations were moderately polydisperse, with PDI values between 0.15 and 0.35, and R11-loaded NPs were associated with the lowest PDI values. In addition, all NPs were associated with partially negative zeta potential values between −20 and −24 mV, consistent with previous reports [26]. Based on the loading efficiency results, R7 NP2 and R11 NP2 (Figure 2) were chosen for further evaluation.

**Figure 3:**
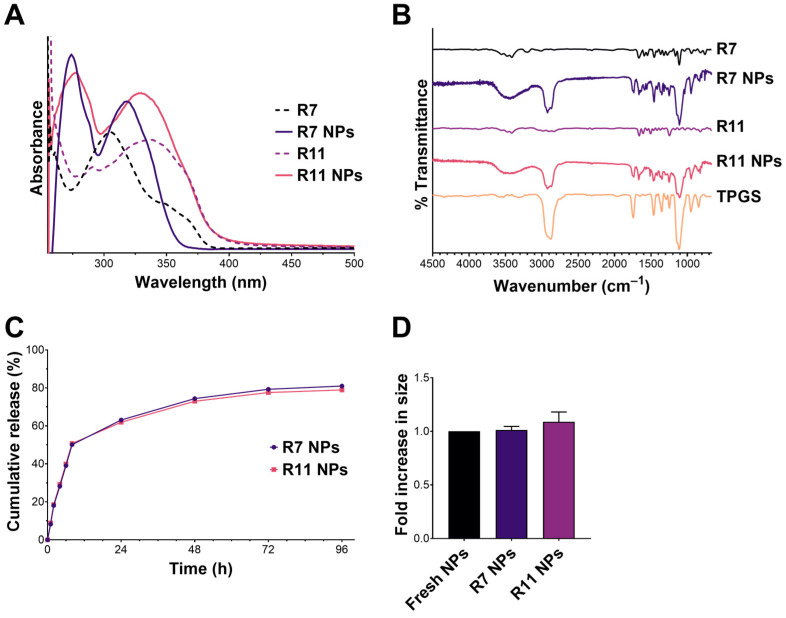
Characterization of R7 and R11 NPs by (**A**) UV–Vis and (**B**) FT-IR spectroscopy; (**C**) In vitro release of R7 and R11 from their respective NPs in PBS pH 7.4, 37 °C (mean ± SD; *n* = 3); (**D**) Stability of R7 and R11 NPs upon incubation with RPMI 1640 medium supplemented with 10% FBS for 1 h at 37 °C. Results are expressed as the fold increase in size relative to fresh NPs (mean ± SD; *n* = 3).

The UV–Vis spectra of R7 and R11 and their loaded NPs are shown in Figure 3A. R7 and R11 displayed absorbance maxima (λ_max_) at 305 nm and 335 nm, respectively. Upon loading in TPGS NPs, λ_max_ for R7 underwent a redshift to 317 nm, and a new peak appeared at 274 nm, which is attributed to TPGS. In the case of R11, λ_max_ underwent a blueshift to 328 nm, and the same TPGS peak appeared at 274 nm. These shifts in λ_max_ may have resulted from intermolecular interactions (e.g., hydrogen bonding and hydrophobic interactions) between the drug molecules and TPGS. The FT-IR spectra of both R7 and R11 (Figure 3B) exhibited a weak –OH stretching band between 3700 and 3300 cm^−1^ and an amide stretching band at 1670 cm^−1^. As for TPGS, the spectrum showed characteristic bands corresponding to C–H, O–C=O, and C–O–C stretching vibrations at 2800–3000, 1750, and 1100 cm^−1^, respectively. Drug-loaded NPs displayed the same characteristic bands as TPGS, in addition to the amide stretching bands of R7 and R11 at 1670 cm^−1^. We also noted more intense –OH stretching in both R7- and R11-loaded NPs, which may have resulted from hydrogen bonding interactions with TPGS in line with UV–Vis findings. As for drug release, R7 and R11 showed almost superimposable release profiles characterized by a biphasic pattern typical of polymeric NPs (Figure 3C). The two NP formulations exhibited a relatively fast release phase during the first 8 h (0–50% cumulative release), followed by a more sustained release phase up to 96 h (50–82% cumulative release). Moreover, the NPs displayed excellent colloidal stability upon incubation with serum-supplemented cell culture medium with minimal changes in particle size (Figure 3D). Collectively, the characterization results confirmed that entrapping R7 and R11 in TPGS NPs by nanoprecipitation is a viable formulation approach that could improve their biological performance.

### 3.2. Antiproliferative Activity of R7- and R11-Loaded NPs

**Figure 4:**
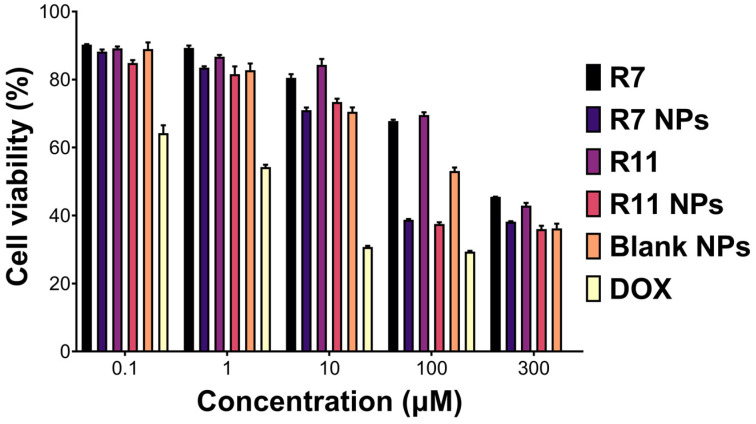
Viability (mean ± SD; *n* = 5) of MDA-MB-231 cells upon treatment with the free compounds R7 and R11, their corresponding NPs, blank (drug-free) NPs diluted to equivalent concentrations as the drug-loaded NPs, and doxorubicin HCl (DOX) for 72 h.

One of the main aims of this study was to evaluate R7 and R11 against TNBC and whether the TPGS-based NP formulation could enhance their anticancer activities. The free drugs and the NPs were tested against MDA-MB-231 cells as a representative TNBC cell line. Cells were incubated with different concentrations of the free drugs, loaded NPs, and blank (drug-free) NPs for 72 h, followed by conducting a viability assay. All treatments exhibited a dose-dependent decrease in cell viability with varying degrees (Figure 4). The most notable differences were apparent at 100 µM (equivalent to the free drugs), where R7 and R11 NPs were significantly more potent than the free compounds. Nonlinear regression analysis was employed to derive the half-maximal inhibitory concentration (IC_50_) corresponding to each treatment. Accordingly, the treatments could be ranked in terms of potency as follows: R7 NPs (20 µM) > R11 NPs (30 µM) > blank NPs (38 µM; equivalent to the drug concentration) > R7 (280 µM) > R11 (290 µM). DOX was employed as a positive control and exhibited an IC_50_ of 1.8 µM. Consequently, entrapping R7 and R11 in TPGS NPs led to a ~10-fold increase in potency, which could in part be attributed to the inherent anticancer activity of TPGS as the blank NPs, which were tested at the same concentration as the loaded NPs exhibited notable antiproliferative activity. Note that the compounds have previously been validated as selective inhibitors of the PI3K/AKT/mTOR pathway by in vitro and in silico methods [19]. Moreover, TPGS NPs were tested in human dermal fibroblasts as a model normal cell line and exhibited limited toxicity [26]. Thus, it is expected that R7 and R11 NPs will be highly selective to cancer cells compared to normal cells.

**Figure 5:**
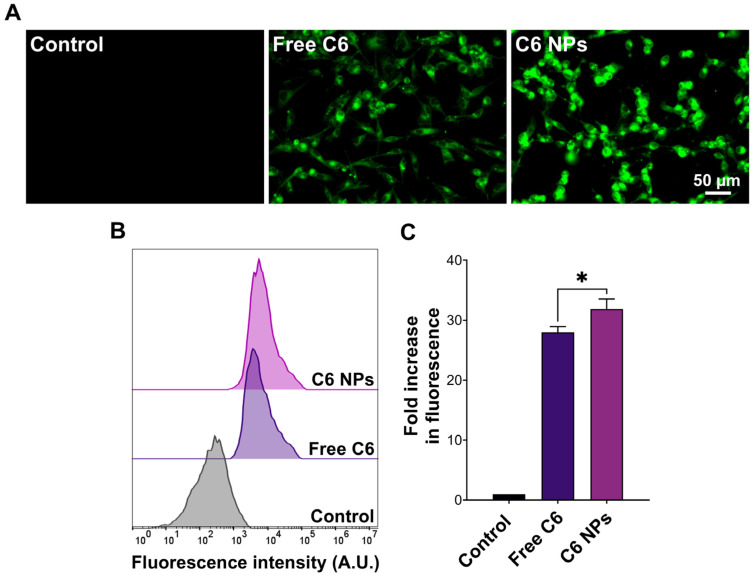
Uptake of C6-labeled NPs by MDA-MB-231 cells. (**A**) Fluorescence microscopy images and (**B**) flow cytometry histograms of cells incubated with free coumarin 6 (C6) or an equivalent concentration of C6-loaded NPs for 1 h compared to the control; (**C**) Cell-associated fluorescence obtained from flow cytometry measurements expressed as the fold increase in fluorescence intensity compared to the control (mean ± SD; *n* = 3; * *p* < 0.05).

The superior anticancer activity of the NPs compared to the free drugs could have also resulted from NP-mediated cellular uptake. To test this hypothesis, the drugs were replaced with C6, and cellular uptake was visualized by fluorescence microscopy. As presented in Figure 5A, C6-loaded NPs were characterized by more intense intracellular green fluorescence signals compared to the free dye. These observations were further corroborated by flow cytometry, where cells incubated with C6-loaded NPs showed significantly greater cell-associated fluorescence (*p* < 0.05) compared to free C6 (Figure 5B,C). The results were aligned with our previous findings, where TPGS/Pluronic P123 NPs entrapping a similar compound were more readily taken up by MCF-7 and MDA-MB-231 cells [26]. TPGS-modification of polymeric and lipid NPs was also reported to enhance cellular internalization of loaded cargo [36,37]. Altogether, the NP formulation could potentiate the anticancer activity of R7 and R11 by inhibiting cancer cell proliferation and facilitating cellular uptake.

### 3.3. Proapoptotic Activity of R7- and R11-Loaded NPs

**Figure 6:**
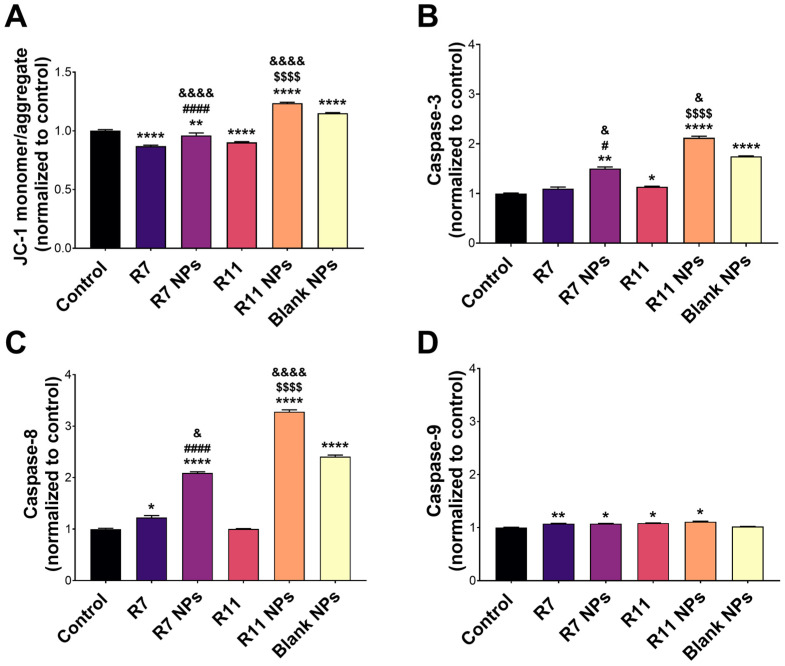
Proapoptotic activity of the free compounds R7 and R11, their corresponding NPs, and blank (drug-free) NPs in MDA-MB-231 cells. (**A**) JC-1 mitochondrial membrane potential assay results expressed as the JC-1 monomer/aggregate ratio normalized to the control (untreated) cells (mean ± SD; *n* = 3); (**B**) Activity of caspase-3, (**C**) caspase-8, and (**D**) caspase-9 upon treating the cells with IC_50_ concentrations of each material for 24 h. Results are expressed as the expression level of each caspase normalized to the control (untreated) cells (mean ± SD; *n* = 3). * *p* < 0.05, ** *p* < 0.01, and **** *p* < 0.0001 compared to the control; ^#^ *p* < 0.05 and ^####^ *p* < 0.0001 comparing R7 NPs and free R7; ^$$$$^ *p* < 0.0001 comparing R11 NPs and free R11; ^&^ *p* < 0.05 and ^&&&&^ *p* < 0.0001 comparing R7 NPs and R11 NPs to blank NPs.

**Figure 7:**
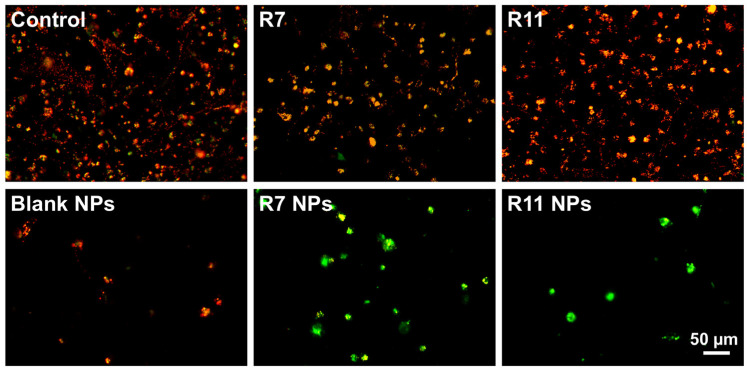
Merged red and green fluorescence microscopy images of MDA-MB-231 cells stained with JC-1 following treatment with IC_50_ concentrations of R7 and R11, their corresponding NPs, and blank (drug-free) NPs for 24 h.

To investigate the potential mechanism of causing cell death, the ability of the developed NPs to induce apoptosis in MDA-MB-231 cells was investigated by conducting a mitochondrial membrane potential assay. Mitochondrial membrane potential can be probed by staining the cells with JC-1, a fluorescent dye that emits red fluorescence when confined within intact mitochondria and green fluorescence upon cytoplasmic release due to the loss of mitochondrial membrane potential during early apoptosis [38]. Thus, the ratio of green/red fluorescence of stained cells serves as an indicator of apoptosis induction. As presented in Figure 6A, R7 exhibited a small but significant decrease in the JC-1 monomer/aggregate ratio compared to untreated cells, indicating a limited ability to induce apoptosis through a mitochondrial-dependent pathway. Although it was also lower than the control, the ratio associated with R7 NPs was significantly higher than that corresponding to free R7 (*p* < 0.0001), which suggests a slight improvement in inducing apoptosis via the mitochondrial pathway. Treating cells with free R11 caused a significant decrease in the JC-1 monomer/aggregate ratio compared to the control, similar to R7. However, when cells were treated with R11 NPs, the JC-1 monomer/aggregate ratio was significantly higher than the control (*p* < 0.0001), and this increase was greater than that obtained from the blank NPs (*p* < 0.0001). These results demonstrate that entrapment of R11 in the NPs improved its ability to induce apoptosis through a mitochondrial-dependent pathway, which was likely enabled by the NP vehicle. The quantitative results are in line with the fluorescence microscopy images of JC-1-stained cells (Figure 7). Cells treated with R7 and R11 NPs demonstrated predominantly green fluorescence signals corresponding to compromised mitochondria, unlike the cells treated with the free compounds, which showed predominantly red fluorescence signals similar to the control.

The activity of proapoptotic caspases was also examined to gain more insights into the molecular mechanism of inducing cell death by the compounds and their NP formulations. Caspase-3, -8, and -9 are key caspases involved in the apoptosis cascade, where caspase-3 and -9 are mainly associated with the intrinsic apoptosis pathway, and caspase-8 is a key mediator of the extrinsic pathway [39]. As depicted in Figure 6B, caspase-3 activity in R7-treated cells did not differ from the control. However, R7 NPs caused a notable increase in caspase-3 activity (*p* < 0.01 compared to the control and *p* < 0.05 compared to free R7). On the other hand, R11 treatment resulted in a significant increase in caspase-3 activity compared to the control (*p* < 0.05), which was further enhanced by R11 NPs (*p* < 0.0001 compared to the control and free R11). As with the JC-1 assay, the enhancement of caspase-3 activity by R11 NPs was most likely promoted by the NP vehicle itself since the blank NPs demonstrated an increase in caspase-3 activity. Concerning caspase-8 activity (Figure 6C), free R7 was associated with a significant increase compared to the control (*p* < 0.05), unlike R11. Moreover, both R7 NPs and R11 NPs promoted caspase-8 activity more than the free compounds (*p* < 0.0001). In the case of R11 NPs, the increase in caspase-8 activity was notably greater than that achieved with the blank NPs (*p* < 0.0001). In terms of caspase-9 expression, the free compounds and their corresponding NPs exhibited a small yet significant increase compared to the control (*p* < 0.05), with no difference detected between the compounds and their NP formulations, and the blank NPs had no effect on caspase-9 activity (Figure 6D). Our results show that R7 can promote apoptosis by activating both the extrinsic (caspase-8) and intrinsic (caspase-9) pathways, while R11 mainly acts via the intrinsic apoptosis pathway involving caspase-3 and -9. Importantly, entrapping the compounds in the NPs significantly improved their proapoptotic activity via both pathways, as the NP vehicle itself could increase the expression of caspase-3 and -8.

### 3.4. Effect of R7- and R11-Loaded NPs on MDA-MB-231 Spheroid Formation

**Figure 8:**
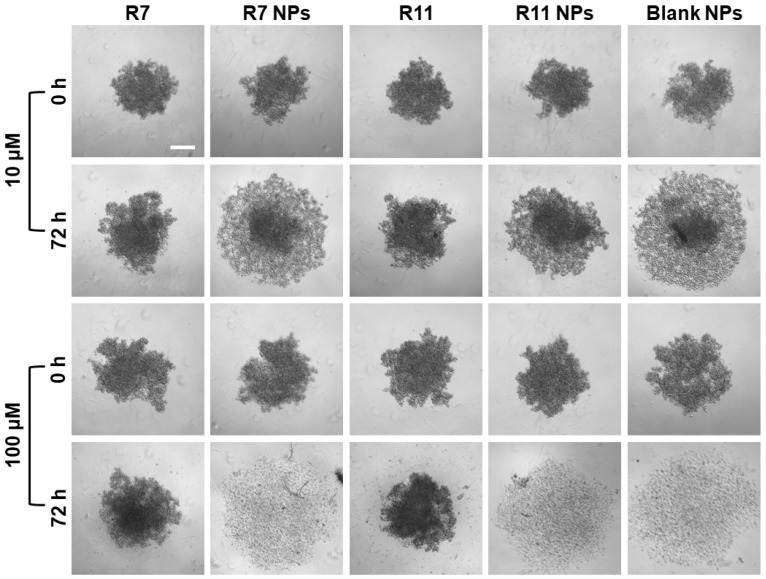
Effect of R7- and R11-loaded NPs on multicellular MDA-MB-231 spheroids compared to the free compounds and blank NPs. The spheroids were imaged at 0 and 72 h after treatment with 10 and 100 µM of each material (scale bar = 200 µM).

Multicellular tumor spheroids have emerged as an important in vitro tool to study the anticancer effect of various agents as spheroids more closely resemble the three-dimensional cellular organization of the tumor tissue in vivo [40]. Thus, they can help predict the in vivo effectiveness of chemotherapeutic agents and their formulations, including their cytotoxicity and tumor penetration ability. In this study, the effect of R7 and R11, as well as their NP formulations on multicellular spheroids of MDA-MB-231 cells, was evaluated by monitoring the morphological changes occurring as a result of each treatment. The cells were allowed to proliferate in ultra-low attachment plates for 72 days, resulting in the formation of spheroids around 500 µm in diameter. The spheroids were imaged before treatment (t = 0 h) and then treated with low and high concentrations (10 and 100 µM) of each compound, the drug-loaded NPs, and the blank NPs for 72 h. The spheroids were imaged again and compared to their original shape and size. Morphological features that are most commonly evaluated in multicellular tumor spheroids include their size/diameter, sphericity, and compactness [41]. As shown in Figure 8, MDA-MB-231 cells tended to form spheroids that were somewhat irregular in shape, but the most distinguishing morphological feature was their compactness before and after treatment. In the case of the free compounds R7 and R11, they both showed a weak ability to cause loosening of the compact spheroids even at the high concentration (100 µM). Conversely, spheroids treated with the NPs at 10 µM (equivalent to the free drugs) underwent significant loosening and dissociation of the cells forming the spheroid structure. Moreover, upon treatment with the high dose, NP-treated spheroids completely disintegrated, leaving behind a halo of cell debris. These observations were attributed to the NP vehicle itself, which showed comparable effects on spheroid morphology as drug-loaded NPs. Importantly, these findings signify the excellent ability of the NP formulation to facilitate the uptake and tumor penetration of the two compounds.

TPGS is a pharmaceutical excipient commonly used as a permeation enhancer due to its amphiphilic properties as well as biocompatibility [42,43]. In nanomedicine, TPGS is often used to modify various types of NPs, such as those based on polymers and lipids, to improve the loading of lipophilic drugs, overcome biological barriers, and combat multidrug resistance [44,45,46]. The anticancer activity of TPGS has been suggested to be related to its effect on the mitochondrial electron transport chain, eventually leading to apoptosis [47]. It has also been shown to cause downregulation of anti-apoptotic proteins such as survivin and to promote cell cycle arrest in breast cancer cell lines MCF-7 and MDA-MB-231 cells [48]. TPGS-based nanocarriers demonstrated enhanced cellular uptake both in cell monolayers and multicellular tumor spheroids [36,37,49], which translated into improved in vivo targeting and accumulation in tumor tissues [49,50]. These attributes make TPGS an ideal nanocarrier for new anticancer compounds such as R7 and R11, which can improve their biological potency and biopharmaceutical properties.

## 4. Conclusions

Here, we investigated the anticancer activity of two newly synthesized PI3Kα inhibitors, R7 and R11, against TNBC. A TPGS-based NP formulation was developed for the compounds to improve their biological performance and enable future preclinical and clinical investigations. The compounds were successfully entrapped in TPGS NPs by nanoprecipitation, producing particles ranging between 100 and 200 nm in diameter with 70–90% encapsulation efficiency and sustained release. The two NP formulations demonstrated a significant improvement in anticancer activity against MDA-MB-231 cells compared to the free compounds by enhancing cellular uptake and promoting apoptosis. The NPs also facilitated tumor penetration in an in vitro model of multicellular tumor spheroids, emphasizing the promising role of TPGS-based NPs as a potential nanomedicine formulation for new anticancer drug candidates.

## Figures and Tables

**Table 1:** Composition of R7- and R11-loaded NPs.

Sample Code	R7 (mg)	R11 (mg)	TPGS (mg)
R7 NP1	1	-	10
R7 NP2	2	-	10
R11 NP1	-	1	10
R11 NP2	-	2	10

Sample Code

R7 (mg)

R11 (mg)

TPGS (mg)

**Table 2:** Characterization of R7- and R11-loaded NPs (mean ± SD; *n* = 3).

Sample Code	Particle Size (nm)	PDI	Zeta Potential (mV)	Loading Efficiency (%)
R7 NP1	175 ± 10	0.35 ± 0.08	−22 ± 3	70 ± 5
R7 NP2	203 ^a^ ± 12	0.26 ± 0.03	−20 ± 2	76 ± 7
R11 NP1	109 ^b^ ± 6	0.15 ± 0.02	−23 ± 4	84 ± 3
R11 NP2	100 ^c^ ± 4	0.20 ± 0.02	−23 ± 2	91 ± 1
Blank NP	128 ± 7	0.33 ± 0.06	−24 ± 4	-

^a^ *p* < 0.05 compared to R7 NP1; ^b^ *p* < 0.0001 compared to R7 NP1; ^c^ *p* < 0.0001 compared to R7 NP2.

## Data Availability

The authors confirm that all the data supporting the findings of this study are available within the article.
